# Inhibition of Epac1 suppresses mitochondrial fission and reduces neointima formation induced by vascular injury

**DOI:** 10.1038/srep36552

**Published:** 2016-11-10

**Authors:** Hui Wang, William G. Robichaux, Ziqing Wang, Fang C. Mei, Ming Cai, Guangwei Du, Ju Chen, Xiaodong Cheng

**Affiliations:** 1 Department of Integrative Biology and Pharmacology, The University of Texas Health Science Center, Houston, Texas, USA; 2 Texas Therapeutics Institute, The University of Texas Health Science Center, Houston, Texas, USA; 3 The Brown Foundation Institute of Molecular Medicine, The University of Texas Health Science Center, Houston, Texas, USA; 4 Department of Gastrointestinal Surgery, Union Hospital, Tongji Medical College, Huazhong University of Science and Technology, Wuhan, Hubei Province, China; 5 Department of Medicine, University of California, San Diego, La Jolla, California, USA

## Abstract

Vascular smooth muscle cell (VSMC) activation in response to injury plays an important role in the development of vascular proliferative diseases, including restenosis and atherosclerosis. The aims of this study were to ascertain the physiological functions of exchange proteins directly activated by cAMP isoform 1 (Epac1) in VSMC and to evaluate the potential of Epac1 as therapeutic targets for neointima formation during vascular remodeling. In a mouse carotid artery ligation model, genetic knockdown of the Epac1 gene led to a significant reduction in neointima obstruction in response to vascular injury. Pharmacologic inhibition of Epac1 with an Epac specific inhibitor, ESI-09, phenocopied the effects of Epac1 null by suppressing neointima formation and proliferative VSMC accumulation in neointima area. Mechanistically, Epac1 deficient VSMCs exhibited lower level of PI3K/AKT signaling and dampened response to PDGF-induced mitochondrial fission and reactive oxygen species levels. Our studies indicate that Epac1 plays important roles in promoting VSMC proliferation and phenotypic switch in response to vascular injury, therefore, representing a therapeutic target for vascular proliferative diseases.

Cardiovascular disease (CVD) is the leading cause of morbidity and mortality worldwide. CVD is manifested by a range of pathological conditions affecting the heart or blood vessels. Inappropriate vascular smooth muscle cell (VSMC) activation plays an important role in the development of intima hyperplasia associated with atherosclerosis and restenosis^1^,^2^,^3^. However, no clinically effective therapeutic targets for the prevention and treatment of neointima formation have been identified. In response to injury, VSMCs migrate from the tunica media through the damaged endothelia and become hyperproliferative, leading to neointima formation and vessel remodeling^4^,^5^. The transition of VSMC phenotype from contractile to synthetic induced by injuries is characterized by proliferation and extracellular matrix synthesis^6^. Therefore, understanding the signaling mechanism in the activation of VSMCs is critical for the development of novel treatment strategies for vascular proliferative diseases.

Exchange proteins directly activated by cAMP isoform 1 (Epac1) is a guanine nucleotide exchange factor (GEF) under the control of intracellular cAMP, a major stress-response second messenger. Activation of Epac1 by cAMP further triggers down-stream RAS superfamily small GTPases, Rap1 and Rap2, which are critical for a wide variety of biological functions, ranging from cytoskeleton organization and intracellular trafficking to cell adhesion and junction^7^,^8^,^9^. Studies based on genetic Epac1 knockout mice have demonstrated that Epac1 contributes to leptin resistance^10^,^11^, rickettsial infection^12^, chronic pain^13^,^14^, stress induced phospholamban phosphorylation in cardiomyocytes^15^, Treg-mediated immune-suppression^16^, and cardiomyocyte hypertrophy^17^. However, the physiological roles of Epac1 in VSMC function and neointima formation remain controversial^18^,^19^,^20^,^21^,^22^,^23^. Here we show that deletion of Epac1 in mice significantly suppresses neoinitima formation by inhibiting VSMC proliferation in response to vascular injury. This protective effect of Epac1 deficiency is in part mediated by Epac1’s functions in modulating mitochondrial morphology and cellular reactive oxygen species (ROS) activity. Most importantly, pharmacological inhibition of Epac *in vivo* recapitulates Epac1 knockout phenotype, demonstrating the therapeutic efficacy of Epac inhibitors for the treatment of vascular proliferative diseases.

## Results

### Epac1 deficiency inhibits neointima formation after vascular injury

**Figure 1:**
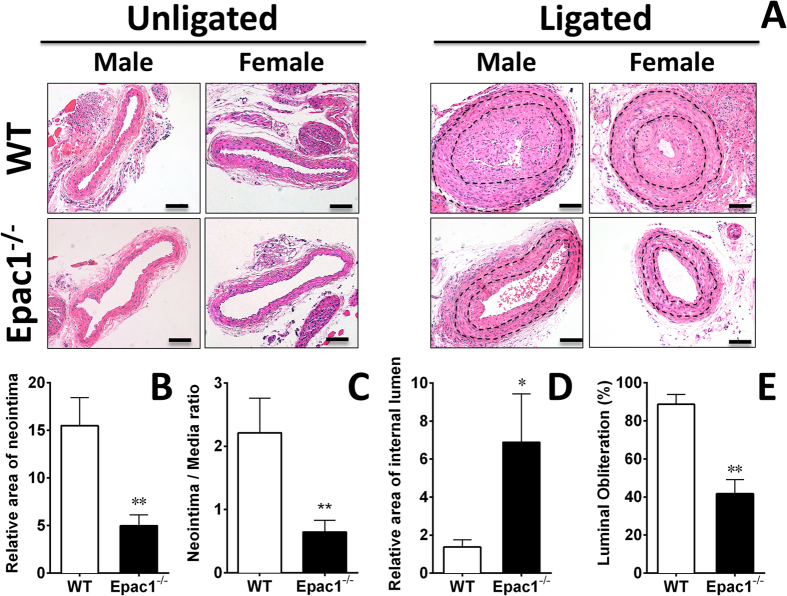
Epac1 knockdown alleviates neointima formation in carotid arteries after ligation *in vivo*. Left side carotid artery from WT (N = 7) and Epac1^−/−^ mice (N = 7) were ligated and analyzed at day 28 after ligation. Luminal obliteration was significantly reduced in the carotid artery from Epac1^−/−^ mice compared to WT mice. (**A**) H&E stained cross sections of contralateral unligated and ligated carotid arteries in WT and Epac1^−/−^ mice. Black lines indicate inner and outer elastic lamina. Quantification of neointima area (**B**) and neointima/media ratio (**C**) of injured carotid arteries from WT and Epac1^−/−^ mice. The lumen inside the external elastic lamina (EEL) was significantly larger in the Epac1^−/−^ group (**D**) while the luminal obliteration, defined as percentage of the neointima taking up the internal elastic lamina, was significantly reduced in the Epac1 null mice. (**E**) Data are expressed as mean ± SEM. N = 7 from pooled male and female samples. *P < 0.05, **P < 0.01.

To ascertain the functional roles of Epac1 in response to vascular injury, we employed a well-characterized carotid artery ligation mouse model to compare neointima formation in WT and Epac1^−/−^ littermates. Histological analysis of injured arteries 28 days after ligation showed that the lumens of the ligated vessels were almost completely blocked in WT controls while Epac1 deficiency led to dramatic reductions in neointima formation (Fig. 1A). Morphometric analysis of injured carotid arteries revealed a significant decrease in intimal area (Fig. 1B), as well as a more than 3-fold reduction in intima/media ratio (Fig. 1C), accompanied with a 5-fold increase in lumen area in Epac1^−/−^ mice as compared to WT controls (Fig. 1D). The luminal obliteration in Epac1^−/−^ mice was significantly reduced when compared with that of WT mice (WT 88.7% vs. Epac1^−/−^ 41.7%) (Fig. 1E). On the other hand, the thicknesses of the tunica media were not significantly different between Epac1^−/−^ and WT groups (Fig. S1). These observations are consistent with a recent study by Kato *et al*.^20^.

**Figure 2:**
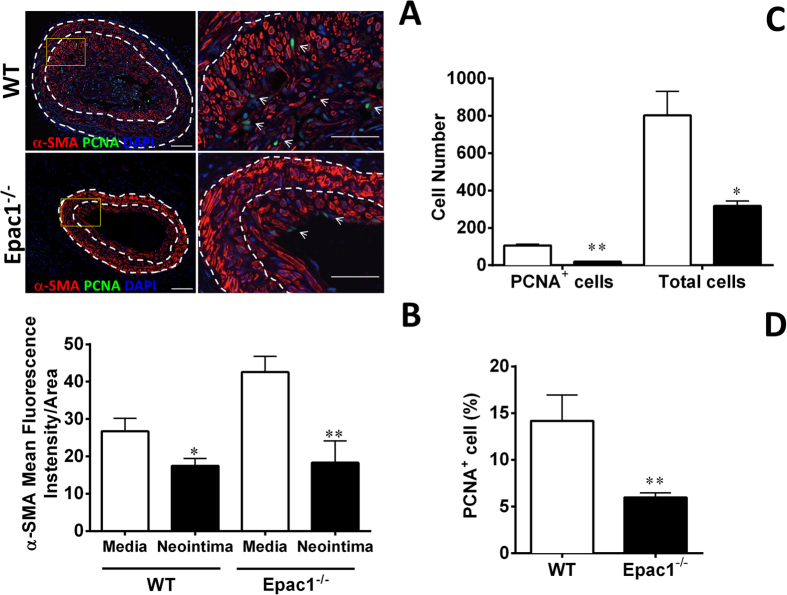
Epac1 deficiency inhibits cell proliferation in neointima. (**A**) Representative immunoflorescent images of injured carotid arteries from WT and Epac1^−/−^ mice stained with anti-α-SMA to visualize VSMCs and PCNA to identify proliferative cells. Cell nuclei are counterstained with DAPI (blue). (**B**) Quantification of the mean fluorescence intensity of α-SMA per area for the tunica media or neointima of WT and Epac1^−/−^ samples shown in A. (**C**) Cell counts of the PCNA positive and total cell number in the neointima of WT (open bars) and Epac1^−/−^ mice (closed bars) after caratoid ligation as shown in A. (**D**) Quantification of the percentage of PCNA positive cells within the neointima of WT and Epac1^−/−^ mice after carotid ligation. Data are expressed as mean ± SEM. N = 4–5. *P < 0.05, **P < 0.01 vs WT control.

While the majority cells within the intima lesion were positive for α-smooth muscle actin (α -SMA) staining, the α-SMA intensity of the VSMC in neointima area was weaker than those in the media region in both WT and Epac1^−/−^ groups (Fig. 2A,B). Furthermore, we observed a significant reduction in the total number and percentage of proliferative cells as indicated by positive PCNA staining in Epac1^−/−^ neointima (Fig. 2C,D). Immunostaining of endothelial cells showed that the endothelial layer was well organized and intact in lesions from Epac1^−/−^ mice while endothelia in WT lesions were disorganized and heavily infiltrated by α-SMA positive VSMCs (Fig. S2).

### Epac1 deficiency inhibits VSMC proliferation and migration *ex vivo*

**Figure 3:**
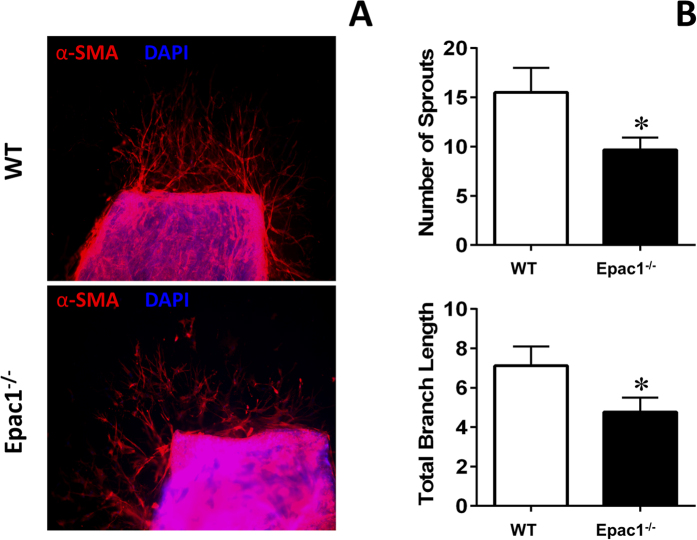
Epac1 is important for vascular smooth muscle cell proliferation *ex vivo*. (**A**) Representative immunoflorescent images of aortic rings from WT and Epac1^−/−^ vessels (red, α-SMA; blue, DAPI). (**B**) Quantification of the images represented in panel A for the number of VMSC sprouts emerging from the aortic ring as well as the total length of all branches for each outgrowth. Data are expressed as mean + SEM. N = 6–8 images per group. *P < 0.05 vs WT control.

VSMC proliferation and migration are fundamental steps contributing to neointima formation in response to vascular injury^24^,^25^. To investigate the cellular mechanism for the protective function of Epac1 deficiency in neointima formation after vessel injury, we isolated aortic explants from WT and Epac1^−/−^ mice to compare the VSMC growth in a 3D collagen matrix containing platelet-derived growth-factor (PDGF), which plays a central role in promoting SMC proliferation and migration^26^,^27^. We found that VSMC outgrowth was significantly reduced in Epac1^−/−^ aortic explants compared with WT aortic explants in response to PDGF. α-SMA immunofluorescent staining of the aortic explants showed that the average number of VSMC outgrowth sprouts and the total length of all branches per explant were significantly more in the WT than those from the KO counterparts (Fig. 3A,B). These *ex vivo* results, consistent with the *in vivo* phenotypic data, suggest that Epac1 deficiency reduces VSMC proliferation and/or migration in response to PDGF stimulation.

### Epac1 deficiency suppresses VSMC proliferation and PI3K/AKT signaling pathway

**Figure 4:**
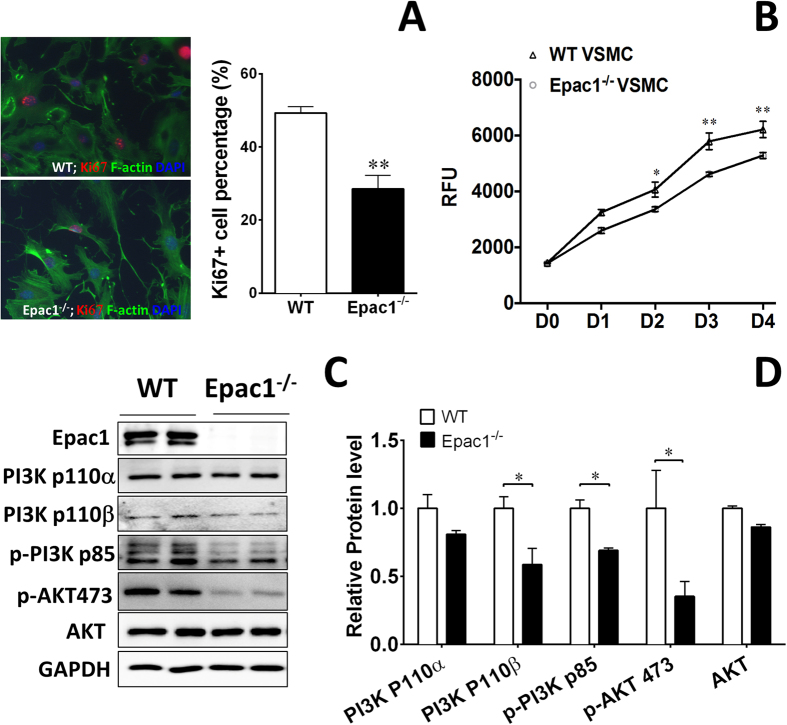
Epac1 deficiency affects the proliferation of VSMC through PI3K/AKT signaling pathway. (**A**) Representative immunoflorescent images of WT and Epac1^−/−^ VSMCs stained with phalloidin and Ki67 (green, F-actin; red, Ki67; blue, nuclei) and quantification of these data as percentage of Ki67 positive cells in WT and Epac1^−/−^ VSMCs. (**B**) Cell proliferation assays of WT and Epac1^−/−^ primary VSMCs grown in culture medium. VSMCs were seeded at 2000 cells/well in 96 well plates in triplicate and cell proliferation was determined by Alamar Blue assay. (**C**) Representative images of the cellular levels of PI3K 110α, PI3K 110β, pPI3K p85, pAKT S473 and total AKT as determined by Western blotting. (**D**) Quantification of protein levels where total PI3K and AKT levels were normalized using loading control GAPDH and p-AKT levels were normalized using total AKT levels. Data are expressed as mean ± SEM. N = 4. *P < 0.05, **P < 0.01 vs WT control.

To further determine the role of Epac1 in VSMC proliferation *in vitro*, we isolated primary aortic VSMCs from WT and Epac1^−/−^ mice and compared the percentage of Ki67 positive VSMCs *in vitro*. As shown in Fig. 4A proliferative Ki67 positive population was significantly reduced in Epac1^−/−^ VMSCs. Cell proliferation analyses using Alamar-Blue assay further confirmed that growth of Epac1-deficient VSMCs was significantly reduced (Fig. 4B). Among the known signaling pathways mediating the proliferative effect of PDGF, the activation of MAPK pathway was not affected by Epac1 deletion (Fig. S3). On the other hand, the level of PI3K/AKT signaling in Epac1^−/−^ VSMC were decreased as compared to WT VSMCs (Fig. 4C,D). Altogether, these data indicate that Epac1 impacts PDGF-induced VSMC proliferation via modulating PI3K/AKT signaling, which is known to play key roles in the phenotypic switch of VSMCs^28^,^29^,^30^.

### Epac1 deficiency reduces PDGF-induced mitochondrial fission and reactive oxygen species (ROS) production

**Figure 5:**
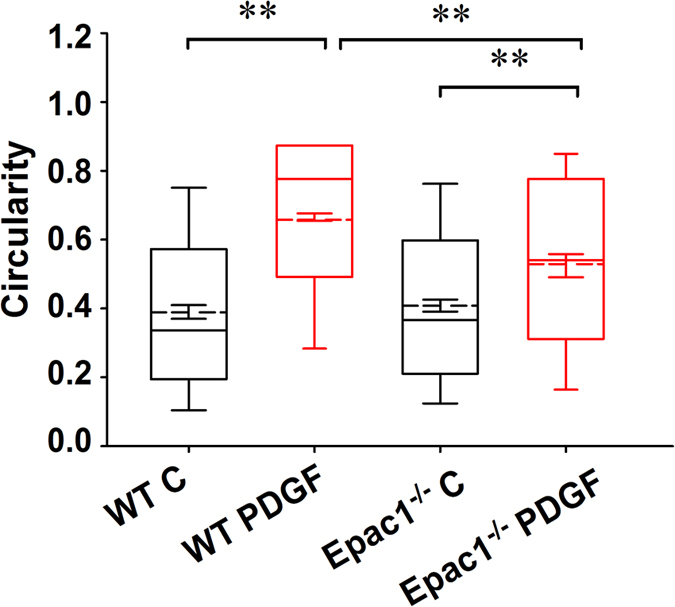
Epac1 mediates PDGF-induced mitochondrial fission. Changes in mitochondria fission as measured by alteration in mitochondrial circularity in WT and Epac1^−/−^ VSMCs in response to PDGF (10 ng/ml) stimulation. As depicted in the box and whiskers plot, the boundaries of the box represent the 25th to 75th percentiles, whereas the solid whiskers represent the 10^th^ to 90^th^ percentiles in the dataset. The solid line represents the median and the dashed line represents the mean value for each group. Error bars within the box depict ±SEM for each group. Number of mitochondria particles detected by the analysis program were as follows: WT unstimulated (3340), WT PDGF (11386), KO unstimulated (3954), KO PDGF (2802).

Mitochondrial fission and ROS production induced by PDGF are known cellular triggers for proliferation/migration of VSMCs during vascular remodeling^31^. Epac1 contains a mitochondrial targeting sequence and has been shown to co-localize with mitochondria^32^. Subcellular fractionation analysis and live cell co-localization imaging of Epac1-GFP and MitoTracker confirmed Epac1’s localization to mitochondria in VSMC (Fig. S4). To evaluate the role of Epac1 in mitochondrial function of VSMCs, we examined mitochondrial morphology of the VSMCs. While mitochondrial circularity distribution of WT and Epac1^−/−^ VSMCs were comparable at the basal state with mean circularity measurement at 0.39 (WT) and 0.41 (Epac1 null), mitochondrial circularity was dramatically increased in response to PDGF stimulation in WT VSMCs, suggesting mitochondrial fragmentation/fission. In contrast, changes in mitochondrial morphology were significantly less in the Epac1^−/−^ VSMCs in response to PDGF treatment as compared with the WT PDGF treated group (Figs 5 and S5).

**Figure 6:**
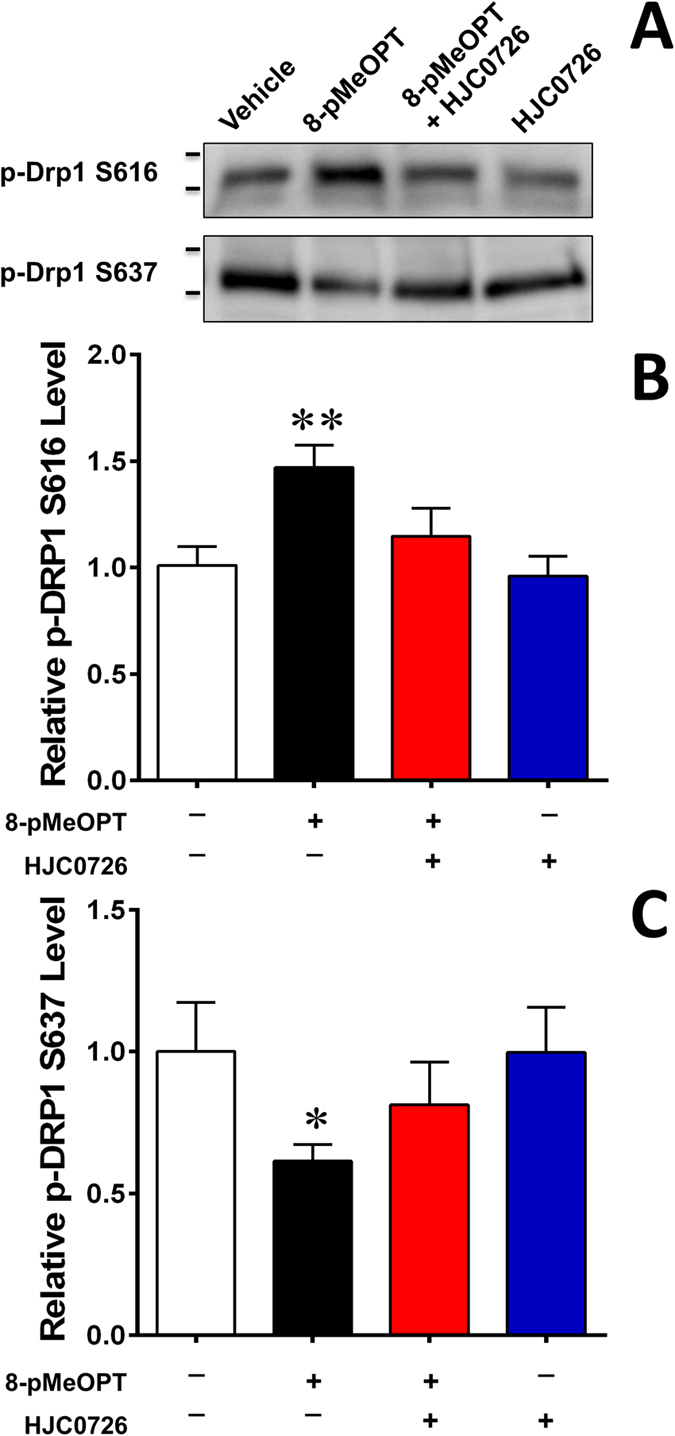
DRP1 phosphorylation at Ser616 and Ser637 are inversely modulated by Epac1 activation. (**A**) Representative immunoblot of DRP1 phosphorylation levels at serine 616 and 637 after pharmacological activation/inhibition of Epac1 in rat VSMCs with 8-pMeOPT-2′-O-Me-cAMP (100 μM, 24 h) or HJC0726 (3 μM, 30 min pretreatment) respectively. Activation of Epac1 exhibits opposing effects on the two phosphorylation sites. (**B**) Quantification of p-DRP1 Ser616 and p-DRP1 Ser637 was determined by immunoblot, where total protein was used as the loading control. Data are expressed as mean ± SEM. N = 6. *P < 0.05, **P < 0.01 vs WT control.

To determine if Epac1 is directly involved in regulating dynamin-related protein (DRP1, also known as dynamin-1-like protein, DLP1) phosphorylation, which plays a critical role in mitochondrial dynamics, we monitored the levels of DRP1 phosphorylation at serine 616 and 637 sites in VSMCs responding to stimulation of an EPAC-specific cAMP analog, 8-(4-Methoxyphenylthio)-2′-O-methyladenosine-3′,5′-cyclic monophosphate (8-pMeOPT-2′-O-Me-cAMP). As shown in Fig. 6, activation of Epac in VSMCs led to an upregulation of phosphor-Ser616, a fission-promoting phosphorylation site, and a downregulation of phosphor-Ser637, a fission-suppressing phosphorylation site. The effects of 8-pMeOPT-2′-O-Me-cAMP on DRP1 phosphorylation could be largely suppressed by co-treatment of VSMC with an Epac-specific inhibitor, HJC0726^33^. Taken together, these results suggest that Epac1 promotes mitochondrial fission by modulating DRP1 phosphorylation.

Mitochondrial fission has been implicated in the production of reactive oxygen species (ROS)^34^,^35^, which are known to induce VSMC proliferation in response to PDGF stimulation^36^. To determine if decreased mitochondrial fission in Epac1^−/−^ VSMCs is associated with altered ROS levels, we monitored the cellular ROS levels in WT and Epac1^−/−^ VSMCs. As shown in Fig. S6, PDGF stimulation led to an increased ROS level in WT VMSCs as expected, but this PDGF-induced increase was abolished and slightly reversed in Epac1^−/−^ VSMCs. These results suggest that Epac1 plays an important role in PDGF-induced ROS production in VSMCs.

### Epac specific inhibitor ESI-09 reduces neointima formation *in vivo*

**Figure 7:**
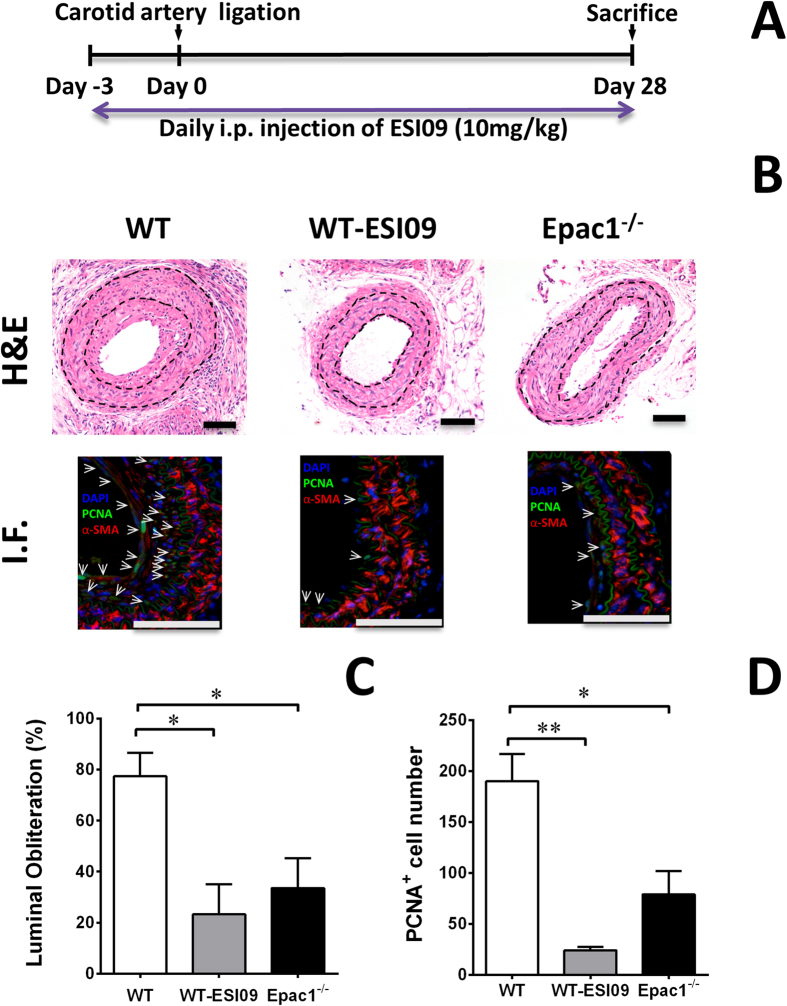
Pharmacological inhibition of Epac1 is sufficient to prevent neointima formation in carotid arteries after ligation *in vivo*. (**A**) Experiment design for the ESI-09 treatment. (**B**) H&E and immunofluorescence images of aortic sections after carotid artery ligation from WT mice treated with ESI-09 or vehicle control, as well as from Epac1 null mice. (**C**) Quantification of lumen obliteration of injured carotid arteries from female WT (vehicle or ESI-09 treated) and Epac1^−/−^ mice. (**D**) Quantification of number of PCNA positive cells present in the neointima of injured carotid arteries from female WT (vehicle or ESI-09 treated) and Epac1^−/−^ mice. Data are expressed as mean ± SEM. N = 3–5. *P < 0.05, **P < 0.01 vs WT control.

To validate the potential of Epac1 as a therapeutic target for vascular proliferative diseases, we treated mice that had undergone carotid artery ligation with an Epac specific inhibitor, ESI-09^33^,^37^,^38^. Female WT C57BL/6 mice were treated with a daily dose of ESI-09 at 10 mg/kg (i.p) for 3 d before and until the end of the experiment. The dosage was based on previous published literature and shown to be safe and effective^10^,^12^,^14^. Female WT and Epac1^−/−^ mice treated with vehicle were used as negative and positive controls, respectively (Fig. 7A). 28 d after carotid artery ligation injury, WT female mice developed significant neointima formation and vessel lumen obstruction. Importantly, WT mice treated with ESI-09 showed a dramatic decrease in neointima formation as compared to the vehicle controls (Fig. 7B,C). Moreover, ESI-09 treatment phenocopied Epac1 null’s protective effect, suggesting the observed inhibitory effects were specific to Epac1. Immunostaining analysis further revealed a significant reduction in PCNA positive VSMCs in neointima lesion for ESI-09 treated arteries (Fig. 7B,D). Altogether, these results demonstrate that ESI-09 is effective in preventing neointima formation in response to vascular injury.

## Discussion

Epac1 is abundantly expressed in heart and the vascular system. While the roles of Epac1 in heart and cardiomyocytes have been extensively investigated^15^,^17^,^39^,^40^, the physiological functions of Epac1 in VSMCs are less studied and remain controversial. On one hand, studies based on primary rat VSMC suggest that Epac1 promotes VSMC migration and positively contribute to neointimal formation^18^,^19^,^20^. On the other hand, Epac activation has also been shown to inhibit VSMC migration and proliferation^21^,^22^ and reduce neointima formation^22^,^23^. In the present study, we define the role of Epac1 in VSMC cell proliferation and migration during neointima formation and evaluate the suitability of Epac as a target for therapeutic intervention for proliferative vascular diseases using both genetic and pharmacological approaches. Our studies show that genetic deletion of Epac1 leads to a dramatic decrease in neointima formation without affecting medial layer thickness in response to vascular injury induced by carotid artery ligation in mouse. In addition to a significant reduction of overall and PCNA positive VSMCs accumulation in the lumen of Epac1^−/−^ arteries, the neointima lesions in Epac1 null mice are covered by an intact layer of endothelia while the structure of endothelia is disorganized in the WT controls. These data indicate that deletion of Epac1 in mice suppresses neointima formation while improves overall vessel integrity. Our results are consistent with a recent report by Ishikawa and colleagues using a mouse femoral artery wire injury model^20^. The fact that artery neointima formation is significantly attenuated in Epac1 null mice in two different murine models of arterial restenosis validates Epac1 as an important therapeutic target for restenosis.

PDGF signaling plays a critical role in VSMC transition from contractile to synthetic phenotype and promotes neointima formation during vascular remodeling^27^,^41^. In Epac1^−/−^ VSMCs, PI3K/AKT signaling, one of the canonical PDGF downstream effectors, is down-regulated, consistent with the reduced neointima formation phenotype in Epac1^−/−^ mice. Epac1 is a positive regulator of the PI3K/AKT pathway^42^, which, along with the down-stream target mTOR, is known to drive neointima VSMC proliferation^43^. Drug-eluting stent (DES) coated with mTOR inhibitor rapamycin is a clinically effective approach in reducing the rates of restenosis associated with neointima hyperplasia^44^. Collectively, our study identifies that Epac1, by acting as an important modulator of the PI3K/AKT signaling cascade, promotes VSMC proliferation during neointima formation. In addition to Epac1’s role in VSMC proliferation, an alternative mechanism by which Epac1 may contribute to neointima formation by promoting VSMC migration has been reported^18^,^20^.

Several lines of evidence strongly support a role for mitochondria fission in the proliferation and phenotypic switch of VSMCs^45^ and in the development of neointima^46^,^47^. Several growth factors, including PDGF increase mitochondrial activity in VSMCs. Mitochondria fission promotes mitochondrial energetics, ROS generation, cell proliferation^48^ and migration^46^. Epac1 contains a mitochondria targeting sequence and has been found to associate with mitochondria^32^,^49^. Consistent with these observations, Epac1 is observed to localize in VSMC mitochondria by subcellular fractionation and live cell imaging studies. Importantly, deletion of Epac1 in VSMC abolishes PDGF-induced mitochondrial fragmentation and ROS generation. Mitochondria morphology is regulated by a set of dynamin-related GTPases: DRP1 for fission, and Mfn1/2 and OPA1 for fusion of the outer/inner mitochondrial membrane, respectively. DRP1 phosphorylation at different sites contributes to the mitochondrial fission differently. Phosphorylation at serine 616 increases mitochondrial fission whereas phosphorylation at serine 637 decreases fission^50^. Treatment with mdivi-1, a pharmacological inhibitor of DRP1, attenuated pulmonary artery hypertension through inhibiting pulmonary VSMC proliferation^45^. Similarly, mdivi-1 inhibited PDGF-induced mitochondrial fragmentation and abolished cell proliferation^48^. Results from this study suggest Epac1 may contribute to mitochondrial dynamics by modulating DRP1 phosphorylation. Previous investigations also depict the protective nature of decreasing ROS production in neointima formation^46^,^51^,^52^. In line with these reports, loss of Epac1 blocked PDGF-induced mitochondrial fission and ROS production and conferred protective phenotype in neointima formation in response to vascular injury.

Our studies demonstrate that Epac1 contributes to neointima formation by promoting PI3K/AKT signaling and mitochondrial fission in VSMC, therefore Epac1 is a potential therapeutic target for vascular proliferative diseases. Moreover, pharmacological studies using a bioactive Epac-specific inhibitor, ESI-09, show that ESI-09 treatment recapitulates Epac1 null phenotype and is sufficient to prevent neointima formation induced by vascular injury. Thus, these data validate Epac inhibition as an effective treatment for vascular proliferative diseases. Furthermore, small molecule Epac inhibitors can be developed and formulated as potential anti-proliferative agents in DES for the prevention of restenosis.

## Methods

### Reagents

Antibodies specific against AKT (#9272), phosphor-AKT (S473) (#9271), Epac1 (#4155) and were purchased from Cell Signaling Technology, while anti α-SMA (A5228), Penicillin-streptomycin solution (P4333), Collagen type I, Rat Tail, approximately 3.65 mg/ml, Opti-MEM™ I Reduced Serum Media and Dulbecco’s modified Eagle’s medium (DMEM) (D5030) were obtained from Sigma Aldrich. Alexa Fluor^®^ 488 Phalloidin (A12379), Alexa Fluor 488 anti-Rabbit IgG (A-11008), MitoTracker and ProLong Antifade Mountant with DAPI (P36935) were purchased from Life Technologies (Carlsbad, CA, USA). Recombinant human PDGF-BB (100–14B) was purchased from PeproTech (Rocky Hill, NJ, USA). Collagenase II and Elastase were purchased from Worthington Biochemical (Lakewood, NJ, USA). The EPAC selective agonist 8-pMeOPT-2′-O-Me-cAMP were purchased from BioLog Life Science Institute (Bremen, Germany).

### Experimental animals

Epac1^−/−^ mice were generated and back-crossed for more than 12 generations to C57BL/6 background (Charles River Laboratories, Wilmington, MA) as described previously^10^. Animals were housed on a 12/12 h light-dark cycle in virus-free facilities with free access to food and water. Use of all animals was in accordance with protocols approved by the Institutional Animal Care and Use Committee of the University of Texas Health Science Center at Houston. All methods were performed in accordance with the relevant guidelines and regulations.

### Mouse carotid artery ligation-injury model

10-week old female and male C57BL/6 WT and Epac1^−/−^ littermates were used. The mouse carotid artery ligation model was described previously^53^. Briefly, the left common carotid arteries of Epac1^−/−^ and wild type mice were exposed and ligated. Four weeks after the surgery, all animals were anesthetized and perfused with PBS and 10% Formalin *in situ*. The ligation-injured segments and contralateral non-injured carotid arteries were collected for evaluation.

### Histology and lesion quantification

Vessels were fixed with 10% formalin, dehydrated and embedded in paraffin. To keep consistency between mice, we collected the tissue slides by section 100 μm from the ligated site. Hematoxylin/eosin staining was performed. HE stained carotid artery cross sections were measured and averaged from at least two representative stained tissue sections (5 μm thick) at least 50 μm apart per carotid artery. The medial area was calculated by subtracting the area defined by the internal elastic lamina (IEL) from the area defined by the external elastic lamina, and the neointima area was calculated as the difference between the area inside the IEL and the luminal area. The luminal obliteration was defined as the percentage of area within the IEL blocked by the neointima. Image-Pro Plus Software was used for measurement.

### Immunofluorescence staining

Heat-induced antigen retrieval with citric antigen retrieval solution (Sigma) was performed in a steamer for 20 min. Sections were blocked with 5% normal goat serum and 1% BSA in PBS for 30 min. Primary antibodies of following sources were used: α-SMA (ab5694), calponin (CALP), PCNA, BS-lectin conjugated with FITC. Corresponding secondary antibody was conjugated to Alexa Fluor 488 or Alexa Fluor 647 (Invitrogen). Sections were incubated with secondary antibodies for 1 h at 37 °C, followed by cell nucleus staining with DAPI for 10 min. Imaging was performed using a Nikon A1 confocal imaging system. Analysis was carried out with Nikon NIS-Elements software.

### *Ex vivo* mouse aortic ring culture and quantification

The mouse aortic ring assay was performed as described previously^54^. In brief, fresh thoracic aortae were harvested from WT and Epac1^−/−^ mice and placed in in sterile MEM buffer. After removing periadventitial fat and connective tissues, the aortae were sliced into ring segments approximately 0.5 mm in thickness. The aortic rings were rinsed and fasted overnight in MEM buffer before embedding into collagen matrix. The aortic rings were randomly divided and individually placed into 96-well plate with each well coated with 50 μl type I collagen matrix (1 mg/ml in DMEM) on ice. At least 20–25 rings per group were used for each experiment. The 96-well plate was placed at room temperature for 15 min and then incubated 1 hour in a humidified incubator (37 °C, 5% CO_2_). Subsequently, PDGF (10 ng/ml) in 150 μl Opti-MEM culture medium was added into each well. The explants were maintained in a humidified incubator (37 °C, 5% CO_2_) with PDGF-containing medium replaced every other day. After 3–5 days, outgrowths of VSMCs were observed and imaged using an inverted microscope.

Digital images of WT and Epac1^−/−^ aortic ring sections were processed using ImageJ software. A convolve filter was applied to each image to track the outgrowth network from the ring. Each outgrowth emerging directly from the ring was identified (number of sprouts), traced, and branch points marked. ImageJ segmented line tool was then used to measure the longest continuous branch, sum length of all branches (total branch length), and furthest distance from the ring for each outgrowth.

### Isolation of mouse VSMC

For mouse primary VSMC isolation, mouse aorta was dissected and digested with 1 mg/ml type 2 collagenase (Worthington) for 10 min. The adventitia was peeled off, and endothelial layer was removed by gently rubbing with sterile cotton-tipped applicator. The media were cut into small pieces and digested in 1 mg/ml collagenase + 0.5 mg/ml elastase for 30 min to acquire single cell suspension. VSMCs were grown in Dulbecco’s Modified Eagle’s Medium (DMEM) containing 10% FBS in a humidified incubator (37 °C, 5% CO_2_), and used for the experiments between passage 3 to passage 4.

### Cell proliferation assay of VSMCs

To assess potential effects on cell proliferation, the Alamar blue assay was performed as described previously^55^. VSMCs were seeded in 96-well tissue culture plates at a low density of about 2000 cells/well, and monitored over a 96-h time frame. Alamar blue (10% vol/vol) was added and further incubated for 6 h before fluorescence was measured.

### Mitochondrial fission analysis

Aortic smooth muscle cells were plated on a poly-lysine coated cover slip and allowed to adhere overnight. The following day culture media was removed and serum-free DMEM was added for 18 h. After serum deprivation, cells were treated with 20 ng/ml of PDGF or vehicle control for 30 minutes (37 °C, 5% CO_2_). Mitochondria were stained using MitoTracker Red for an additional 30 minutes (37 °C, 5% CO_2_). After incubation, cells were washed with sterile PBS twice and the coverslip mounted with PBS for live cell imaging. A minimum of 15 fields of view were captured in this manner for each condition. Quantification of Mitochondria Morphology was accomplished using NIS-Elements Software (Nikon). For each treatment group, mitochondria were counted and analyzed for circularity (4π*Area/Perimeter^2^).

### Cellular ROS measurement

The levels of cellular ROS were detected using a fluorescent probe dihydroethidium (DHE) (Molecular Probes) following manufacturer’s instructions. Cells were loaded with 5 μM DHE at 37 °C for 30 mins. Images were acquired at room temperature and fluorescence intensity was measured and quantified using Image J software.

### Western Blot analysis

Cells were lysed in 1 × SDS buffer supplemented with protease inhibitors and homogenized by sonication. 5–10 μg of protein lysates were subjected to Western blot analyses using antibodies against specific target proteins. The signals were visualized with a Bio-Rad ChemiDoc^TM^ Touch Imaging System using an enhanced chemiluminescence (ECL) kit. Immunoblot images were quantified using Image Lab software (Bio-Rad Laboratories) where each band intensity was determined and normalized to a respective loading control protein immunoblot or total protein (equally sized 1.37 mm segment down center of each lane).

### Statistical analysis

All values are expressed as Mean ± SEM. Intergroup differences were appropriately assessed by either unpaired two-tailed Student’s t-test or one-way analysis of variance (ANOVA).

## Additional Information

**How to cite this article**: Wang, H. *et al*. Inhibition of Epac1 suppresses mitochondrial fission and reduces neointima formation induced by vascular injury. *Sci. Rep*. **6**, 36552; doi: 10.1038/srep36552 (2016).

**Publisher’s note:** Springer Nature remains neutral with regard to jurisdictional claims in published maps and institutional affiliations.

## Supplementary Material

Supplementary Information
