# Supplementary material for: Inhibition of Epac1 suppresses mitochondrial fission and reduces neointima formation induced by vascular injury
Source: Sci Rep. 2016 Nov 10;6:36552. doi: 10.1038/srep36552 (PMC5103196; doi:10.1038/srep36552)
Supplement: Supplementary Information [file srep36552-s1.pdf]

# Inhibition of Epac1 suppresses mitochondrial fission and reduces neointima formation induced by vascular injury

Running title: *Epac1 as a therapeutic target for vascular disease*

Hui Wang[1,2,3]; William G. Robichaux III[1,2,3]; Ziqing Wang[1]; Fang C. Mei[1,2,3]; Ming Cai, [1,4]; Guangwei Du[1]; Ju Chen[5] and Xiaodong Cheng[1,2,3,*]

[1]Department of Integrative Biology and Pharmacology, [2]Texas Therapeutics Institute, [3]The Brown Foundation Institute of Molecular Medicine, The University of Texas Health Science Center, Houston, Texas, USA; [4]Department of Gastrointestinal Surgery, Union Hospital, Tongji Medical College, Huazhong University of Science and Technology, Wuhan, Hubei Province, China, [5]Department of Medicine, University of California, San Diego, La Jolla, California, USA

<u>*To whom correspondence should be addressed</u>:

Xiaodong Cheng, PhD.

Department of Integrative Biology and Pharmacology, The University of Texas Health Science Center, 6431 Fannin Street, Houston, TX 77030-1051

Tel.:713-500-7487

Fax: 409-500-7456

E-mail: xiaodong.cheng@uth.tmc.edu.

**Key Words:** Restenosis; Epac1; therapeutics; neointima; vascular diseases

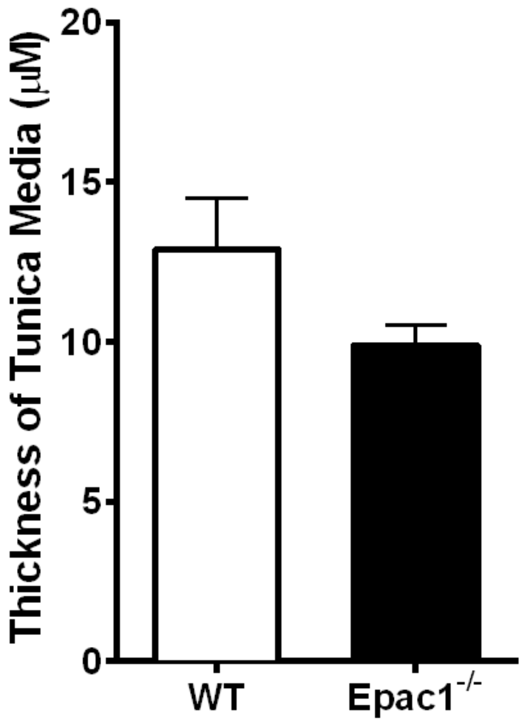


**Supplementary Figure 1. Epac1 deficiency has no effect on the thickness of the tunica media after ligation.** Quantification of neointima area of injured carotid arteries from male WT and Epac1$^{-/-}$ mice. Media area was calculated by area between inner elastic layer and outer elastic layer using Image J software.

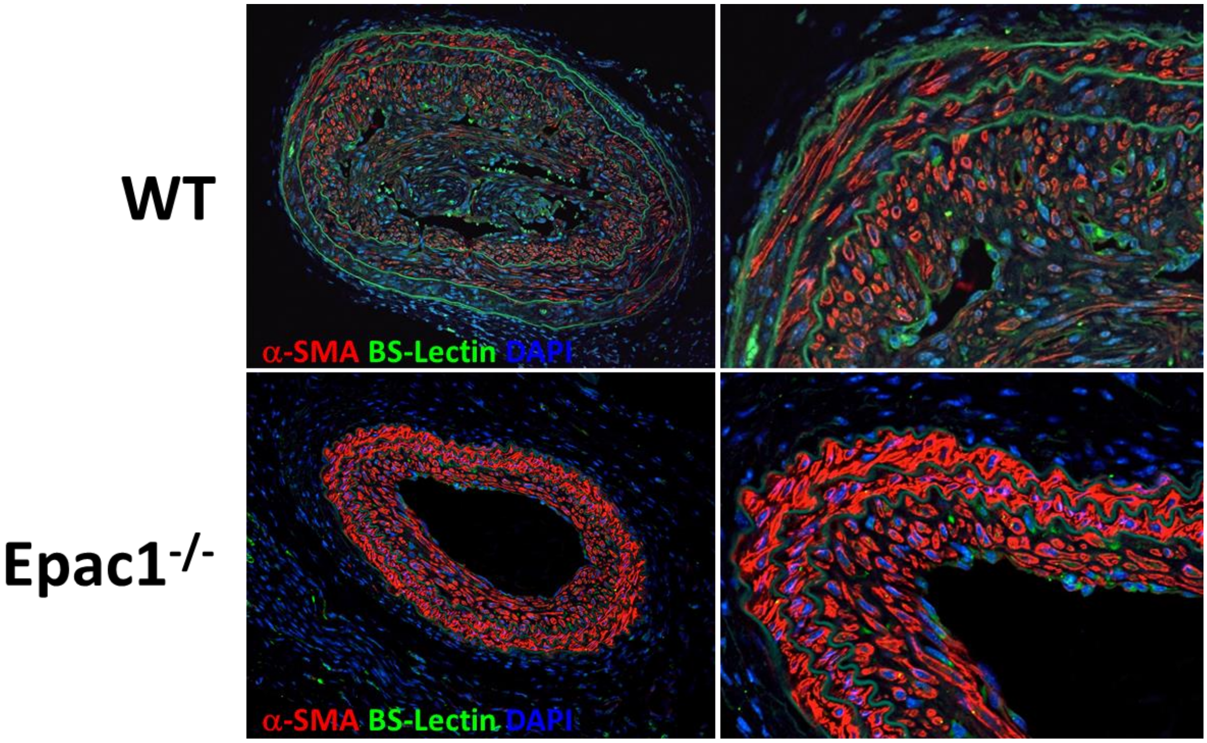


**Supplementary Figure 2. Epac1 deficiency protects the integrity of the endothelial layer during neointima formation in carotid arteries after ligation.** Representative images of injured carotid arteries from WT and Epac1$^{-/-}$ mice stained with BS lectin (green) and α-SMA (red) to identify endothelial cells and VSMCs respectively. Cell nuclei are counter-stained with DAPI (blue).

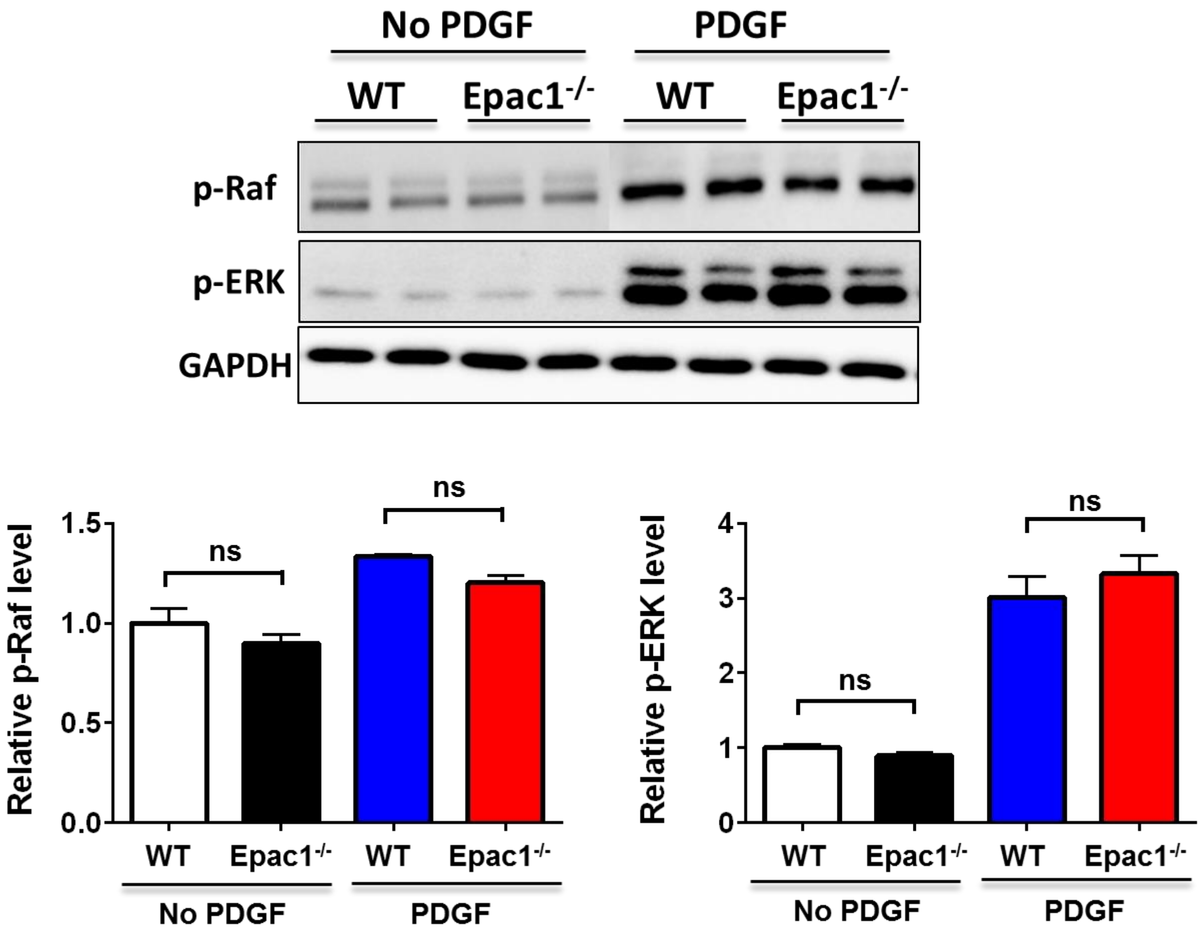


**Supplementary Figure 3. Epac1 deficiency has no significant effect on basal and PDGF-stimulated MAKP signaling.** VSMCs were treated with vehicle control or PDGF for 30 min, and the levels of p-Raf and pERK were determined by Western blotting, and GAPDH was used a loading control.

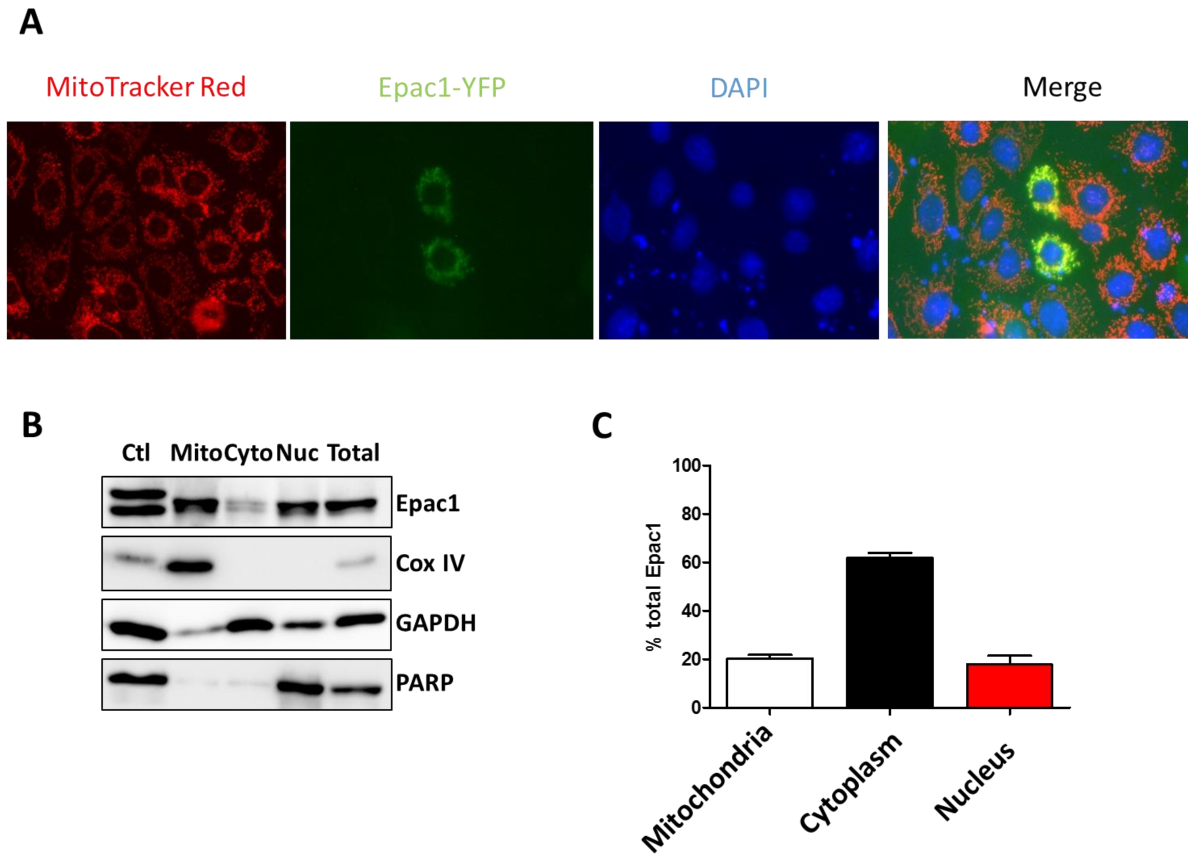


**Supplementary Figure 4. Epac1 localized to mitochondria observed in live cell imaging and cellular fractionation**. (A) Representative images of VSMCs expressing Epac1-YFP (green) stained with MitoTracker Red (red) for mitochondria visualization. Nuclei of cells were counterstained with Hoechst (blue). (B) Representative immunoblot of VSMC cellular fractionation depicting Epac1 expression in mitochondrial (Mito, 1/25 dilution), cytoplasmic (Cyto, 1/300 dilution), and nuclear fractions (Nuc, 1/25 dilution), respectively. Control whole cell lysate from EAhy926 (Ctl) cells and total cell lysate (Total) from VSMC cells (1/300 dilution) were also included. Immunoblots of respective proteins for each cellular fraction were depicted below the Epac1 immunoblot (COX IV – mitochondria, GAPDH – cytoplasm, PARP – nucleus). (C) Densitometry analysis of (B) was conducted using BioRad Image Lab software. Values were multiplied by the fractional dilution (25 for mitochondria and nuclear, 300 for cytoplasm and total cell lysate). The representative level of Epac1 as a percentage of the total cell lysate for each fraction was then determined.

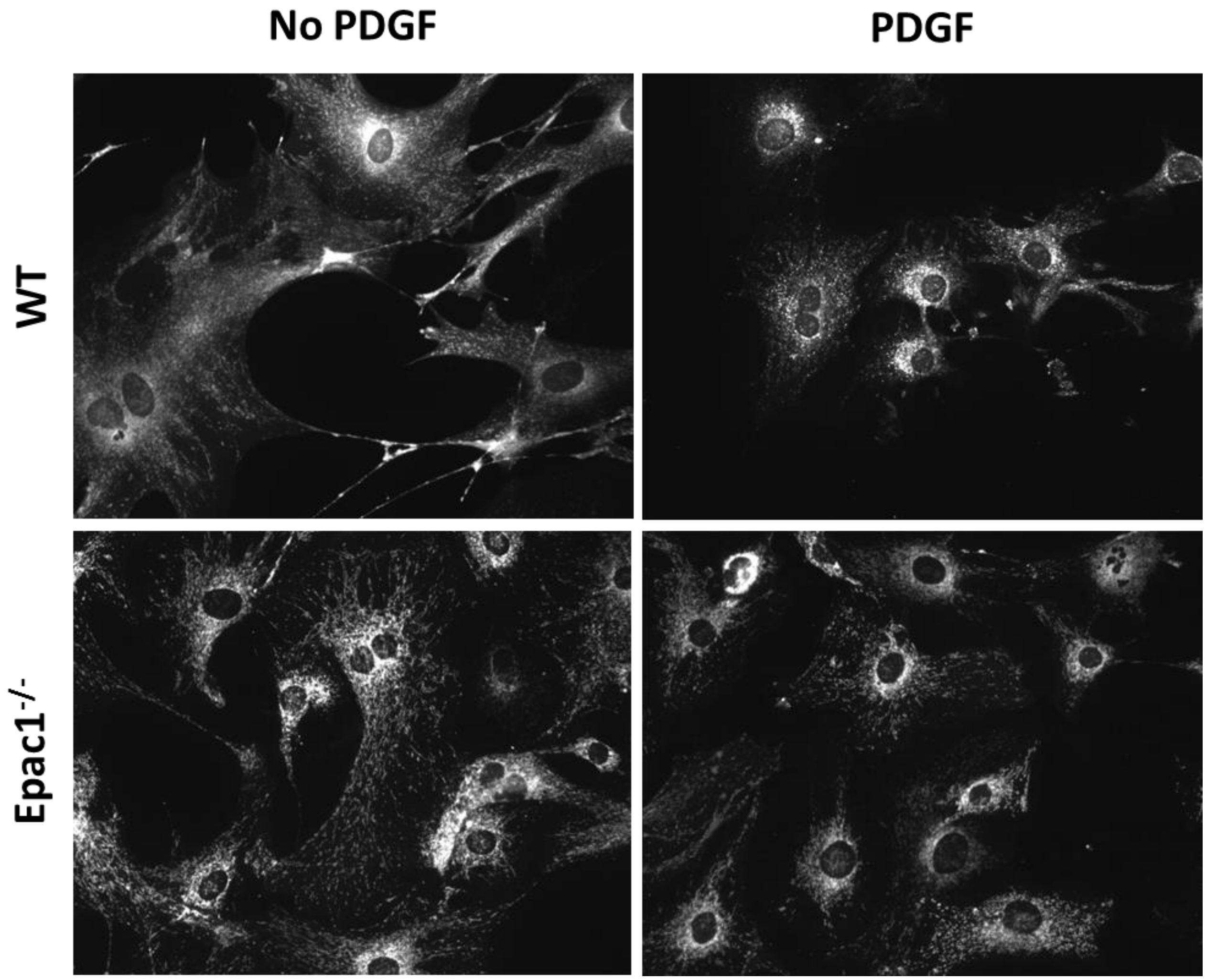


**Supplementary Figure 5. Role of Epac1 in PDGF-induced mitochondrial fission.** Representative pictures for mitochondrial morphology in WT and EPAC1$^{-/-}$ VSMC as visualized with MitoTracker with or without PDGF (10 ng/ml) stimulation.

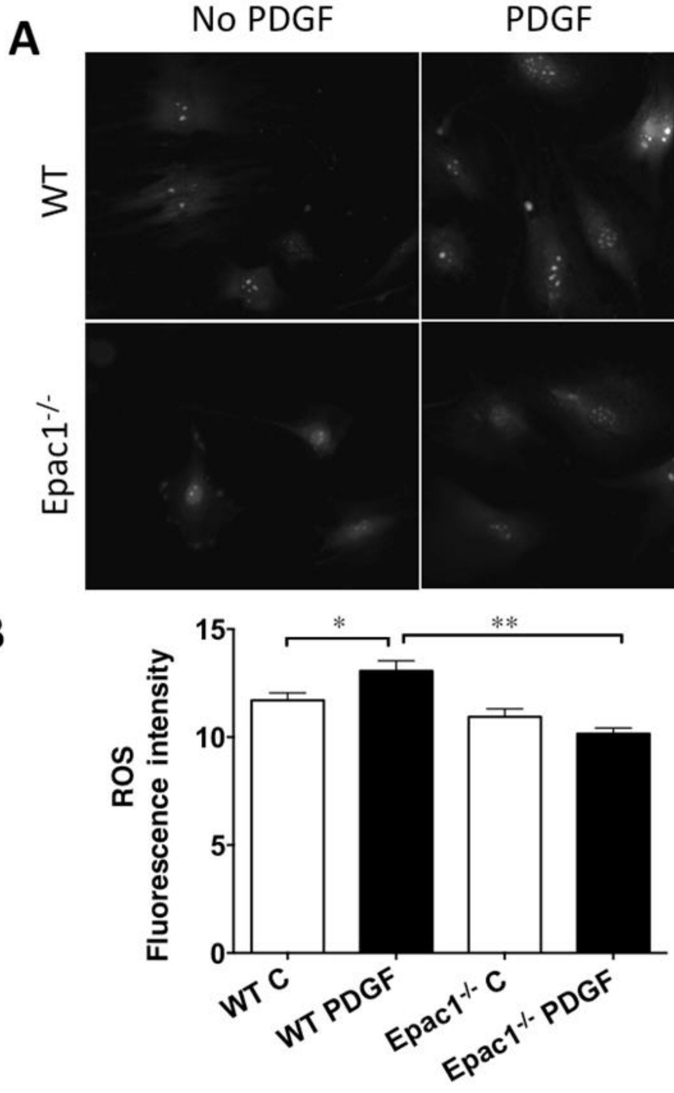


**Supplementary Figure 6. Eapc1 involves in PDGF-induced ROS production in VSMCs.** (A) ROS levels were assessed 30 mins after VSMCs were incubated with 10 ng/ml PDGF. ROS levels were evaluated by DHE in VSMCs. (B) Quantification of fluorescent intensity showed the ROS level was increased after 30 min PDGF stimulation in WT VSMCs, however the response was abolished in Epac1$^{-/-}$ VSMCs. Data are expressed as Mean ± SEM N≥20 cells per group *: $P < 0.05$, **: $P < 0.01$ vs WT control.
